# Analysis of clinicopathological and molecular features of crawling-type gastric adenocarcinoma

**DOI:** 10.1186/s13000-020-01026-7

**Published:** 2020-09-17

**Authors:** Yasuko Fujita, Noriyuki Uesugi, Ryo Sugimoto, Makoto Eizuka, Yosuke Toya, Risaburo Akasaka, Takayuki Matsumoto, Tamotsu Sugai

**Affiliations:** 1 Department of Molecular Diagnostic Pathology, School of Medicine, Iwate Medical University, 2-1-1 Idaidori, Yahaba-cho, Shiwa-gun, Iwate, 028-3695 Japan; 2 Division of Gastroenterology, Department of Internal Medicine, School of Medicine, Iwate Medical University, Yahaba-cho, Shiwa-gun, Iwate, Japan

**Keywords:** Early gastric cancer, Crawling-type adenocarcinoma, *TP53* mutation, Molecular analysis

## Abstract

**Background:**

Crawling-type adenocarcinoma (CRA) is an important gastric cancer (GC) subtype that exhibits a specific histological pattern and has characteristic clinicopathological findings. Despite its characteristic histology, little is known about the molecular characteristics of CRA.

**Methods:**

We examined 177 GC cases, including 51 cases of CRA and 126 cases having conventional differentiated adenocarcinomas (CDAs). Results for immunohistochemistry (mucin phenotype; Muc5AC, Muc6, Muc2 and CD10, CDX-2, MLH-1, p53 and β-catenin), mutation analysis (*TP53*, *KRAS* and *BRAF*), microsatellite instability (BAT25, BAT26, D2S123, D5S346 and D17S250), DNA methylation status by a two-panel method (*RUNX3*, *MINT31*, *LOX, NEUROG1*, *ELMO1* and *THBD*), *MLH-1* promoter methylation, and allelic imbalance (AI; 1p, 3p, 4p, 5q, 8p, 9p, 13q, TP53, 18q and 22q) were examined.

**Results:**

CRAs were more likely to occur in the middle third of the stomach, in younger patients and to be macroscopically depressed. Nuclear accumulation of β-catenin and loss of MLH-1 expression were less frequent among CRA cases compared to CDA cases. At a molecular level, CRA is often characterized by the deletion mutation c.529_546 (18-base pair deletion at codon 177–182 in exon 5) in the *TP53* gene (10 cases). Although the low methylation epigenotype was significantly more frequent for CRAs compared to CDAs, multiple AIs were more often seen in CRAs relative to CDAs.

**Conclusions:**

The results demonstrated that *TP53* mutations, particularly c.529_546del, and multiple AIs are closely associated with CRA carcinogenesis. Our results suggest that CRA is an independent entity of GC in terms of clinicopathologic and molecular findings.

## Background

Gastric cancer (GC) is the fifth most common cancer and the third leading cause of cancer mortality worldwide [1]. According to the World Health Organization (WHO) classification [2], GC comprises five main adenocarcinoma types, including papillary adenocarcinoma, tubular adenocarcinoma, mucinous adenocarcinoma, poorly cohesive carcinoma and mixed adenocarcinoma, as well as other rare entities. The most common histopathological subtype is tubular adenocarcinoma, which can be subclassified into two grades of well- or moderately-differentiated adenocarcinoma [2]. Crawling-type adenocarcinoma (CRA) is an important subtype among moderately-differentiated adenocarcinomas and has attracted increased attention as a specific histological GC subtype due to its characteristic clinicopathological and molecular findings [3–6].

CRA is also referred to as a very well-differentiated gastric carcinoma of the intestinal type [7]. Although cancer glands in CRA show a complex architecture described as a “shaking-hands pattern” or “WHYX pattern” [5, 7], due to subtle cytological atypia such tumor glands can appear to be an “intestinal metaplasia” that is a benign lesion. Therefore, distinguishing this type of GC from non-neoplastic lesions, such as intestinal metaplasia, can be challenging.

In this study we compared the clinicopathologic and molecular features of CRA with those for conventional differentiated adenocarcinoma (CDA). We also aimed to elucidate the clinicopathological features of CRA.

## Methods

### Patients

We retrospectively examined 177 lesions, comprising 51 and 126 CRAs and CDAs, respectively, which were obtained from patients who underwent surgical or endoscopic resection for GC at Iwate Medical University Hospital (Iwate, Japan) between 2010 and 2018. We defined CRA as an adenocarcinoma having branching or anastomosing glands resembling the shapes of the letters W, H, Y or X composed of neoplastic epithelium with low-grade nuclear atypia, as previously proposed [5, 7]. Meanwhile, CDA was defined as an adenocarcinoma that is characterized by tumors having tubular or papillary formations (i.e., CDA corresponds to differentiated-type cancers). The resected specimens were fixed in 10% buffered formalin. After paraffin embedding, representative 3 μm-thick sections were stained with hematoxylin and eosin (H&E) for immunohistochemistry analysis.

The sections were examined by at least two pathologists (T.S. and Y.F.). Histological classification of the tumors in this study was evaluated according to the Japanese classification of gastric carcinoma (the 15th edition) [8] and diagnosis of pTis (dysplasia) and pT1a (intramucosal adenocarcinoma) was made based on the previously described criteria [9].

The study was approved by the Ethical Research Committee of Iwate Medical University (H29–78 and HGH29–17).

### Immunohistochemical studies

Immunohistochemical analysis of Muc2 (Ccp58, dilution 1:100; Leica Biosystems, Nußloch, Germany), Muc5AC (CLH2, dilution 1:100; Leica Biosystems), Muc6 glycoprotein (CLH5, dilution 1:100; Leica Biosystems), CD10 (56C6, ready to use; Agilent Technologies, California, USA), CDX-2 (DAK-CDX2, ready to use; Agilent Technologies), p53 (Dako), β-catenin (β-Catenin-1, ready to use; Agilent Technologies) and MLH-1 (ES05, ready to use; Agilent Technologies) was conducted on 3 μm-thick representative paraffin sections. The DAKO EnVision+ system (dextran polymers conjugated with horseradish peroxidase; DAKO, Copenhagen, Denmark) was used to examine immunohistochemical expression of these markers, as previously described [10].

#### Evaluation of cancer cell phenotypes

Cancer cell phenotypes were subclassified into four groups: gastric type (positive for Muc5AC and/or Muc6, and negative for Muc2 and CD10), intestinal type (negative for Muc5AC and Muc6, and positive for Muc2 and/or CD10), mixed type (positive for Muc5AC and/or Muc6, and also positive for Muc2 and/or CD10) and unclassified type (negative for all markers), according to a previous report [10].

#### Assessment of immunohistochemical expression

To standardize evaluations, we used the following criteria to analyze immunohistochemical staining of mucin markers (MIUC2, MUC5AC, and MUC6), CD10, β-catenin, CDX2, and p53 [11]. The staining intensity scores were divided into four categories: no staining, weak/equivocal staining, moderate staining, and strong staining. Moderate or strong staining was considered to be positive expression. The percentage of cells with positive expression was scored as follows: 0: 0–10% cells; 1: 10% to < 30% cells; 2: 30% to < 60% cells; 3: 60% to < 100% cells; and 4: 100% cells. In this study, a score greater than 1 indicated positive expression of the markers in the lesions. Finally, MLH-1 expression in < 5% of the tumor cell population was defined as loss of MLH-1 expression.

### Tissue dissection and DNA extraction

**Fig. 1:**
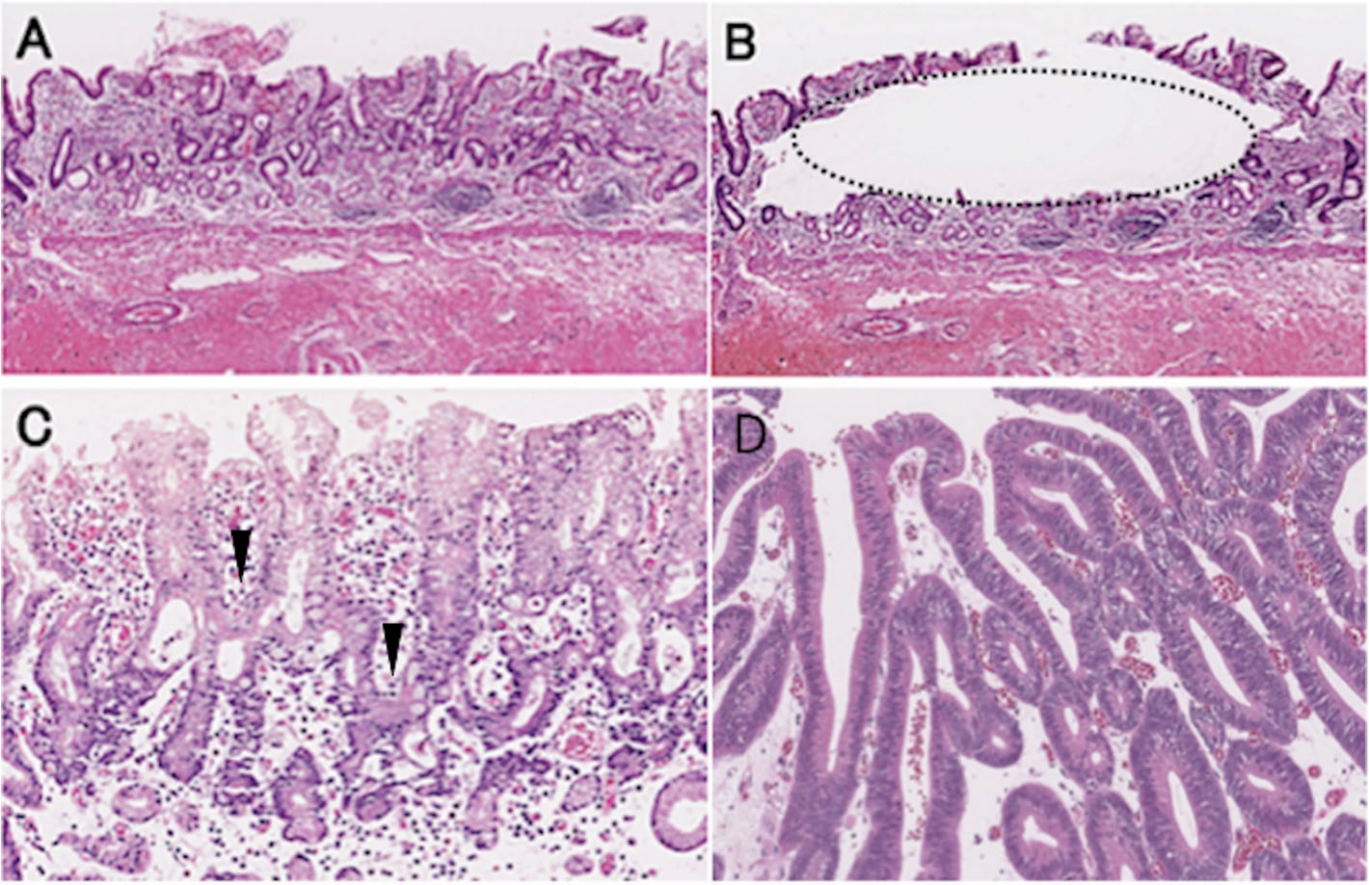
Histology of crawling-type adenocarcinoma (CRA). **a** Irregularly fused glands are seen in the middle upper area of the gastric mucosa at low-power magnification; **b** Section after microdissection and sampling (area enclosed by the dashed line) of CRA for molecular analysis; **c** Cancer glands of CRA showing a “hand-shake” structure (arrow head) with slight cytological atypia at high-power magnification; **d** Representative findings for conventional differentiated adenocarcinoma (CDA)

DNA was extracted from manually micro-dissected paraffin-embedded tissue sliced into 10-μm thick sections (Fig. 1a and b) in which > 60% of cells were identified as tumor cells using TaKaRa DEXPAT (TAKARA Bio Inc., Japan) according to the manufacturer’s instructions.

### Mutation analysis

#### TP53 gene and direct sequence

Single strand conformation polymorphism (SSCP) analysis was performed as previously described, with some modifications [12]. Briefly, the PCR products (2 μl) were mixed with 10 μl gel loading solution (9.5% deionized formamide, 20 mM EDTA-Na, 0.05% xylene cyanol and bromophenol blue), denatured at 95 °C for 5 min and then kept on ice until loading. Non-denaturing 7.5% polyacrylamide gels were used for electrophoresis, which was carried out at 260 to 300 V for 3 to 12 h at 22 °C using a temperature controller (Resolmax, ATTO Co., Tokyo). The gels were visualized by silver staining and photographed.

Sequencing was performed twice on original PCR products of *TP53* exons 5–8 for all SSCP-positive samples to confirm the *TP53* mutation status of the samples using a direct sequence method. The results of the first and second sequencing runs were identical. PCR products were recovered from 3% agarose gels and the eluted DNA fragment was precipitated with ethanol before direct sequencing. Sequence primers were the same as those used for PCR. Direct sequencing was performed using fluorescent-labeled dideoxynucleotide triphosphates for automated DNA sequence analysis (Applied Biosystems 373A sequencer; Applied Biosystems, USA, CA).

#### KRAS and BRAF genes

PCR-pyrosequencing using a PyroMark Q24 instrument (Qiagen, Venlo, the Netherlands) was performed for *KRAS* (exon 2) and *BRAF* (exon 15; codon 600) using a previously reported method [12]. Briefly, the polymerase chain reaction (PCR) product (25 μL) was bound to streptavidin Sepharose HP (GE Healthcare, Brøndby, Denmark), purified, washed, denatured in 0.2 M NaOH and washed again. Before pyrosequencing, 0.3 μM sequencing primer was annealed to the purified single-stranded PCR product by heating to 80 °C for 2 min.

### Analysis of allelic imbalance (AI)

AI analysis was performed using a PCR-microsatellite assay (GeneAmp PCR System 9600; Perkin-Elmer, CA, USA) according to previously reported procedures [10, 12]. AIs on chromosomes 1p, 3p, 4p, 5q, 8p, 9p, 13q, 17p (TP53), 18q and 22q were examined in paired cancer and normal DNA samples using 22 highly pleomorphic microsatellite markers (D1S228, D1S548, D3S1234, D3S2402, D4S1601, D4S2639, D5S582, D5S107, D5S299, D8S201, D8S513, D8S532, D9S171, D9S1118, D13S162, TP53, D18S34, D18S487, D22S274, D22S1140, D22S1168). These markers have frequently been used in studies of GCs [13, 14]. In addition, a variable number of tandem repeat polymorphisms at the *DCC* locus were tested.

PCR reactions were performed using a thermal cycler (GeneAmp PCR System 9600, Perkin-Elmer, CA, USA) with 50–100 ng genomic DNA as a template, 25 pM of each primer, 0.2 mM deoxynucleotide triphosphate (dNTP), 1x reaction buffer containing 1.5 mM MgCl_2_, and 1.5 U *Taq* polymerase (Boehringer Mannheim Co., Germany) in a final reaction volume of 25 μl. Samples were processed for 25 to 30 cycles, with each cycle consisting of 30 s at 94 °C, 1 min at 55 to 58 °C, and 2 min at 72 °C, followed by a final 10 min extension at 72 °C. For quantitative detection of the allelic loss at each locus, PCR-LOH (loss of heterozygosity) analysis was performed as described previously. A 1 μl aliquot of the PCR product was added to 3 μl formamide and 0.5 μl of TAMRA 500 size standard (Applied Biosystems, CA) and was loaded onto a 6% polyacrylamide-8 M urea gel, and run for 2–6 h in a 373A Automated Sequencer (Applied Biosystems, CA, USA) at a constant power of 30 W.

Peaks generated from normal DNA samples were used to determine homozygous (1 peak) or heterozygous (2 peaks). The allelic ratio was calculated as previously described [15]. A cancer was considered to have AI when the allele peak ratio was < 0.60, representing an allelic signal reduction of at least 40%. MSI at a given locus was not evaluated. The data were collected and analyzed using GeneMapper software v. 4.0 (Applied Biosystems, CA, USA).

### Analysis of microsatellite instability (MSI)

The PCR-based assay for evaluation of MSI was described previously [10, 12]. Two adenine mononucleotide repeats (BAT25 and BAT26) and three dinucleotide repeats (D2S123, D5S346 and D17S250) were used to determine the presence of tumor MSI [16]. Tumors were considered positive for MSI when abnormally-sized peaks in the tumor sample relative to the paired normal sample were detected for at least two of the five markers.

### DNA methylation analysis

The DNA methylation status was examined by PCR analysis of bisulfite-modified genomic DNA (EpiTect Bisulfite Kit; Qiagen) using pyrosequencing for quantitative methylation analysis (PyroMark Q24; Qiagen NV). The primers were designed using the PyroMark Assay Design Software package (Qiagen NV). We quantified DNA methylation in 6 specific promoters described by Yagi et al. [17]. High methylation epigenotype (HME) tumors were defined as those having at least 2 methylated markers in the first marker panel (*RUNX3*, *MINT31* and *LOX*). The remaining tumors were screened using a second marker panel (*NEUROG1*, *ELMO1* and *THBD*); intermediate methylation epigenotype (IME) tumors were defined as those having at least 2 methylated markers. The other tumors were designated as having a low methylation epigenotype (LME). Methylation of *MLH-1* was also quantified. The cut-off value was 30% according to a previously described method using six specific promoters [13].

### Statistical methods

Statistical analyses were performed using the statistical computing software R version 3.3.2 (R Foundation for Statistical Computing, Vienna, Austria). To determine significant differences in age and tumor size, the Wilcoxon signed-rank test of variance was used. The Fisher’s exact test was used to compare other clinicopathological factors, immunohistochemical studies and molecular analysis results. Multiple-comparison analysis was carried out using the Bonferroni correction. *P* values < 0.05 were considered to indicate statistical significance.

## Results

### Clinicopathologic features

A total of 51 CRA (Fig. 1c) and 126 CDA lesions were obtained. The clinicopathologic features for CRA and CDA are shown in Table 1.

**Table 1:** Clinicopathologic findings for crawling-type adenocarcinomas (CRAs) and conventional differentiated adenocarcinomas (CDAs)

	CRA (%)	CDA (%)	***P***-value
**Total (lesions)**	51	126	
**Age (years) (median) [range]**	71 [39–86]	73 [45–91]	**0.048**
**Gender**			0.56
Male	35 (68.6)	92 (73.0)	
Female	16 (31.4)	34 (27.0)	
**Location**			**< 0.01**
Upper	7 (13.7)	24 (19.0)	
Middle	32 (62.8)*	46 (36.5)*	
Lower	12 (23.5)*	56 (44.4)*	
**Macroscopic type**			**< 0.01**
Depressed type	37 (72.5)	46 (36.5)	
Elevated, flat or mixed type	14 (27.5)	80 (63.5)	
**Tumor size (mm) (median) [range]**	28 [8–150]	20 [7–103]	**< 0.01**
**Predominant histological component**			**< 0.01**
Well-differentiated adenocarcinoma	8 (15.7)**	100 (79.3)**	
Moderately differentiated adenocarcinoma	41 (80.4)**	19 (15.1)**	
Papillary adenocarcinoma	0 (0.0)	7 (5.6)	
Poorly differentiated adenocarcinoma	2 (3.9)	0 (0.0)	
**Histological subtype**			**< 0.01**
Pure differentiated adenocarcinoma	27 (52.9)	109 (86.5)	
Mixed differentiated and poorly differentiated adenocarcinoma	24 (47.1)	17 (13.5)	
**Tumor extent**			0.68
Mucosa [pTis/pT1a]	34 [0/34] (66.7)	88 [62/26] (69.8)	
Submucosa	17 (33.3)	38 (30.2)	
**Ulcer or ulcer scar**			0.29
Present	12 (23.5)	21 (16.7)	
Absent	39 (76.5)	105 (83.3)	
**Lymphatic involvement**			0.90
Present	6 (11.8)	14 (11.1)	
Absent	45 (88.2)	112 (88.9)	
**Vascular involvement**			0.96
Present	2 (3.9)	3 (2.4)	
Absent	49 (96.1)	123 (97.6)	
**Horizontal margin**			0.08
Positive	8 (15.7)	9 (7.1)	
Negative	43 (84.3)	117 (92.9)	
**Vertical margin**			
Positive	4 (7.8)	14 (11.1)	0.71
Negative	47 (92.2)	112 (88.9)	

*P*-values < 0.05 are in bold text

*, *p* <  0.05; **, p <  0.01

Significant differences in the frequency of tumor locations were seen between CRA and CDA. CRAs were more frequently localized in the middle third of the stomach than were CDAs (CRA, 32/51, 62.8%; CDA, 46/126, 36.5%; *P* <  0.01). Depressed type tumors were significantly more frequent in CRA compared to CDA (37/51, 72.5% vs. 46/126, 36.5%; *P* <  0.01). The median CRA tumor size (28 mm) was larger than that for CDA (20 mm) (*p* <  0.01). The predominant histological component in the CRA group was moderately differentiated adenocarcinoma (41/51, 80.4% vs. 19/126, 15.1%; *P* <  0.01). In addition, the frequency of tumors with a mixed differentiated and poorly differentiated adenocarcinoma component was statistically higher for CRA than for CDA (24/51, 47.1% vs. 17/126, 13.5%; *P* <  0.01). A representative image of histology results for a CRA containing a poorly differentiated adenocarcinoma component is presented in Supplementary Figure 1A-C and shows that this component consisted of poorly cohesive and signet-ring cell carcinoma (SRCC) (Supplementary Figure 1C). Other clinicopathologic variables including gender, presence/absence of ulcer or ulcer scar, lymphovascular invasion and margin status showed no significant differences between CRA and CDA in terms of frequency.

### Immunohistochemical analysis

**Fig. 2:**
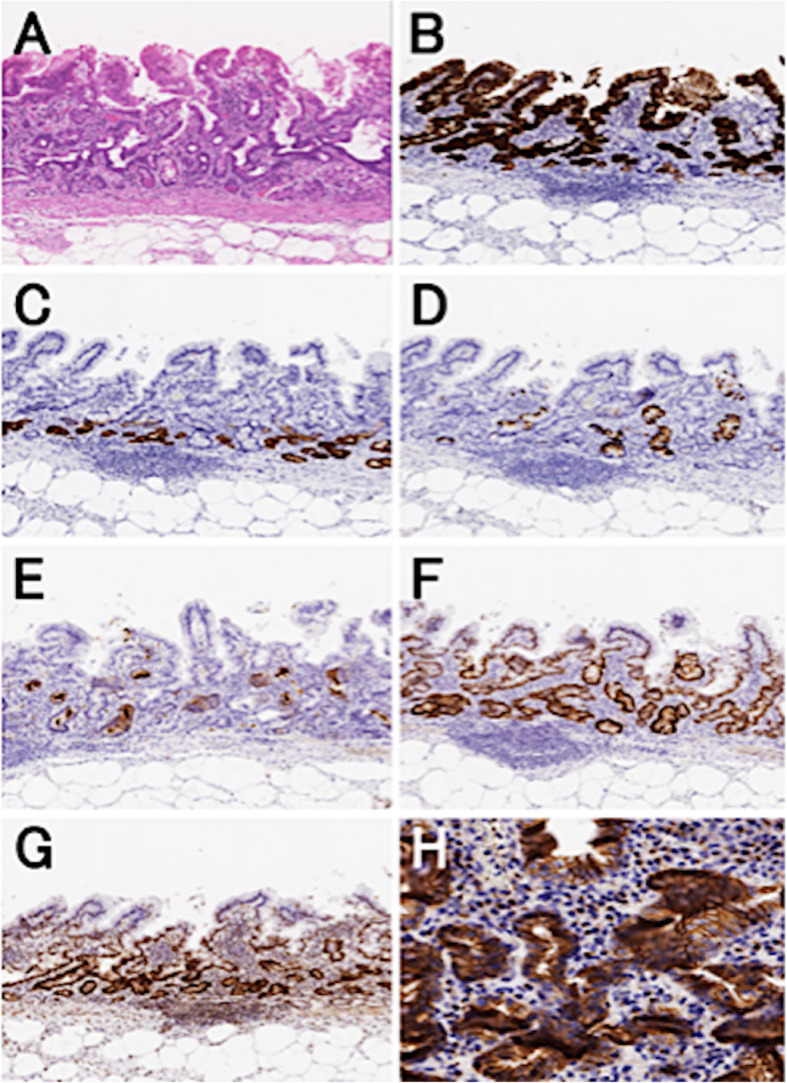
Immunohistochemistry of a sample from a representative case of CRA. **a** Intramucosal crawling-type adenocarcinoma (CRA) can be observed in the hematoxylin and eosin (H&E) section; **b** Cancer glands positive for Muc5AC; **c** focally positive for Muc6 (**d**) and Muc2 (**e**) and negative for CD10. This CRA showed a mixed mucin phenotype and was positive for (**f**) cdx-2. Neither (**g**) loss of MLH-1 expression nor (**h**) β-catenin nuclear accumulation was observed

The immunohistochemistry results are summarized in Table 2. The likelihood of β-catenin nuclear expression was significantly lower in CRAs (1/51, 2.0%) than in CDAs (38/126, 30.3%; *P* <  0.01), as was loss of MLH-1 expression (CRA: 2/51, 3.9%; CDA: 19/126, 15.1%; *P* = 0.04). Meanwhile, no significant differences in the frequencies of mucin phenotype and cdx-2 expression were seen between the two groups. Representative samples are shown in Fig. 2a-h. Finally, there was no statistical difference in the frequency of p53 overexpression between CRA (5/51, 9.8%) and CDA (18/126, 14.3%).

**Table 2:** Immunohistochemical findings for crawling-type adenocarcinomas (CRAs) and conventional differentiated adenocarcinomas (CDAs)

	CRA (%)	CDA (%)	***P***-value
**Total**	51 (100.0)	126 (100.0)	
**Mucin phenotype**			0.59
Gastric type	19 (37.3)	35 (27.8)	
Intestinal type	12 (23.5)	39 (31.0)	
Mixed type	18 (35.3)	48 (38.1)	
Null type (unclassified type)	2 (3.9)	4 (3.2)	
**CDX-2**			0.26
Positive	41 (80.4)	91 (72.2)	
Negative	10 (19.6)	35 (27.8)	
**p53**			0.422
Positive	5 (9.8)	18 (14.3)	
Negative	46 (90.2)	108 (85.7)	
**β-catenin (nuclear accumulation)**			**< 0.01**
Positive	1 (2.0)	38 (30.2)	
Negative	50 (98.0)	88 (69.8)	
**MLH-1**			**0.04**
Retained	49 (96.1)	107 (84.9)	
Loss of expression	2 (3.9)	19 (15.1)	

*P*-values < 0.05 are in bold text

### Molecular analysis

The *TP53* mutation frequency was higher for CRA (19/51, 37.3%) than CDA (10/126, 7.9%; *P* <  0.01) (Table 3) and 10 cases of CRA had the specific *TP53* mutation c.529_546del (18-base pair deletion at codon 177–182 in exon 5, 10 cases), which is a rare mutation in CDAs (Table 4).

**Table 3:** Molecular analysis of crawling-type adenocarcinomas (CRAs) and conventional differentiated adenocarcinomas (CDAs)

	CRA (%)	CDA (%)	***P***-value
**Total (lesions)**	51 (100.0)	126 (100.0)	
***TP53*** **gene mutation**			**< 0.01**
Positive	19 (37.3)	10 (7.9)	
Negative	32 (62.7)	116 (92.1)	
***KRAS*** **gene mutation**			0.09
Positive	0 (0.0)	7 (5.6)	
Negative	51 (100.0)	119 (94.4)	
***BRAF*** **gene mutation**			0.64
Positive	1 (2.0)	0 (0.0)	
Negative	50 (98.0)	126 (100.0)	
**Microsatellite stability status**			0.59
Microsatellite instability (MSI)	5 (9.8)	16 (12.7)	
Microsatellite stable (MSS)	46 (90.2)	110 (87.3)	
**DNA methylation status**^a^			**< 0.01**
High methylated epigenotype	6/45 (13.3)	32 (25.4)	
Intermediate methylated epigenotype	12/45 (26.7)*	64 (50.8)*	
Low methylated epigenotype	27/45 (60.0)**	30 (23.8)**	
**MLH-1 methylation status**			**0.03**
Hypermethylated	1 (2.0)	16 (12.7)	
No hypermethylation	50 (98.0)	110 (87.3)	
**Allelic imbalances**^a^			
1p	17/43 (39.5)	10/93 (10.8)	**< 0.01**
3p	9/37 (24.3)	17/104 (16.3)	0.28
4p	17/36 (47.2)	15/111 (13.5)	**< 0.01**
5q	17/44 (38.6)	31/110 (28.2)	0.21
8p	16/25 (64.0)	20/105 (19.0)	**< 0.01**
9p	10/23 (43.5)	18/103 (17.5)	**< 0.01**
13q	4/12 (33.3)	13/75 (17.3)	0.19
17p (TP53)	10/34 (29.4)	29/94 (30.9)	0.95
18q	21/48 (43.8)	18/112 (16.1)	**< 0.01**
22q	12/23 (52.2)	20/110 (18.2)	**< 0.01**

^a^ Positive number/informative case (%) in DNA methylation status and allelic imbalances

*P*-values < 0.05 are in bold text

*, *p* < 0.05; **, *p* < 0.01

**Table 4:** *TP53* mutations and p53 overexpression in crawling-type adenocarcinomas and conventional differentiated adenocarcinomas

	CRA (%)		CDA (%)	
	*TP53* mutation	p53 overexpression	*TP53* mutation	p53 overexpression
Total cases	51	51	126	126
Cases	19 (37.3)	5 (9.8)	10 (7.9)	18 (14.3)
Number of *TP53* mutation loci	29 (56.9)	–	10 (7.9)	–
**Type of** ***TP*53 mutation**				
**Exon 5**				
c.427G > A (p.Val143Met)	1	0	0	0
c.440 T > A (p.Val147Asp)	1	1	0	0
c.443A > G (p.Asp148Gly)	1	0	0	0
c.446C > T (p.Ser149Phe)	1	0	0	0
c.459C > T (p.Pro153Pro)	1	1	0	0
c.476C > T (p.Ala159Val)	1	0	0	0
c.493C > T (p.Gln165Ter)	1	1	0	0
c.507G > T (p.Met169Ile)	1	0	0	0
c.509C > T (p.Thr170Met)	1	0	0	0
c.529_546del18 (p.Pro177_Cys182del)	10	0	0	0
**Exon 6**				
c.566C > T (p.Ala189Val)	0	0	1	0
c.586_624del39 (p.Arg196_Asp208del)	0	0	1	1
**Exon 7**				
c.722C > T (p.Ser241Phe)	0	0	1	1
c.742C > T (p.Arg248Trp)	2	1	0	0
c.743G > A (p.Arg248Gln)	0	0	2	2
c.776A > G (p.Asp259Gly)	1	0	0	0
**Exon 8**				
c.817C > T (p.Arg273Cys)	1	0	1	1
c.818G > A (p.Arg273His)	4	1	1	1
c.817_818delinsTA (p.Arg273Try)	1	0	0	0
c.839_840delinsCG (p.Arg280Thr)	1	0	0	0
c.844C > T (p.Arg282Trp)	0	0	1	1
c.847_897del51 (p.Arg283_Leu299del)	0	0	1	1
c.853G > A (p.Glu285Lys)	0	0	1	1

*CRA* crawling-type adenocarcinoma; *CDA* conventional-type adenocarcinoma

Of the 19 CRA cases exhibiting *TP53* mutations, 12/19 had deletion mutations that resulted in negative p53 expression (Table 4). The remaining 7 cases had missense mutations, and of these, 5 showed p53 overexpression, indicating that missense mutation of *TP53* correlated with p53 overexpression. For the remaining two cases carrying a *TP53* mutation, no p53 overexpression was detected. Meanwhile, for the 10 cases of CDA with *TP53* mutation, 9 had missense mutations and the remaining case had a deletion type mutation. All 9 CDA cases with a missense mutation exhibited p53 overexpression, yet the other 9 CDA cases without *TP53* mutation (18–9 = 9) also showed p53 overexpression. Thus, these 9 CDA cases were examples of p53 overexpression occurring in the absence of TP53 mutation. The *TP53* mutations are summarized in detail in Table 4. There were no significant differences in the *KRAS* and *BRAF* mutation frequencies between the two groups.

Although there was no significant difference in MSI frequency between the two groups, CRAs were significantly more likely to have a low DNA methylation epigenotype than CDAs (27/45, 60.0% vs. 30/126, 23.8%; *P* <  0.01; Table 3). Meanwhile, the frequency of *MLH-1* methylation was lower for CRAs than CDAs (1/51, 2.0% vs. 16/126, 12.7%; *P* = 0.04; Table 3). Of the 10 different AIs assessed, 6 (1p, 4p, 8p, 9p, 18q and 22q) were more frequent in CRAs than in CDAs (Table 3).

**Fig. 3:**
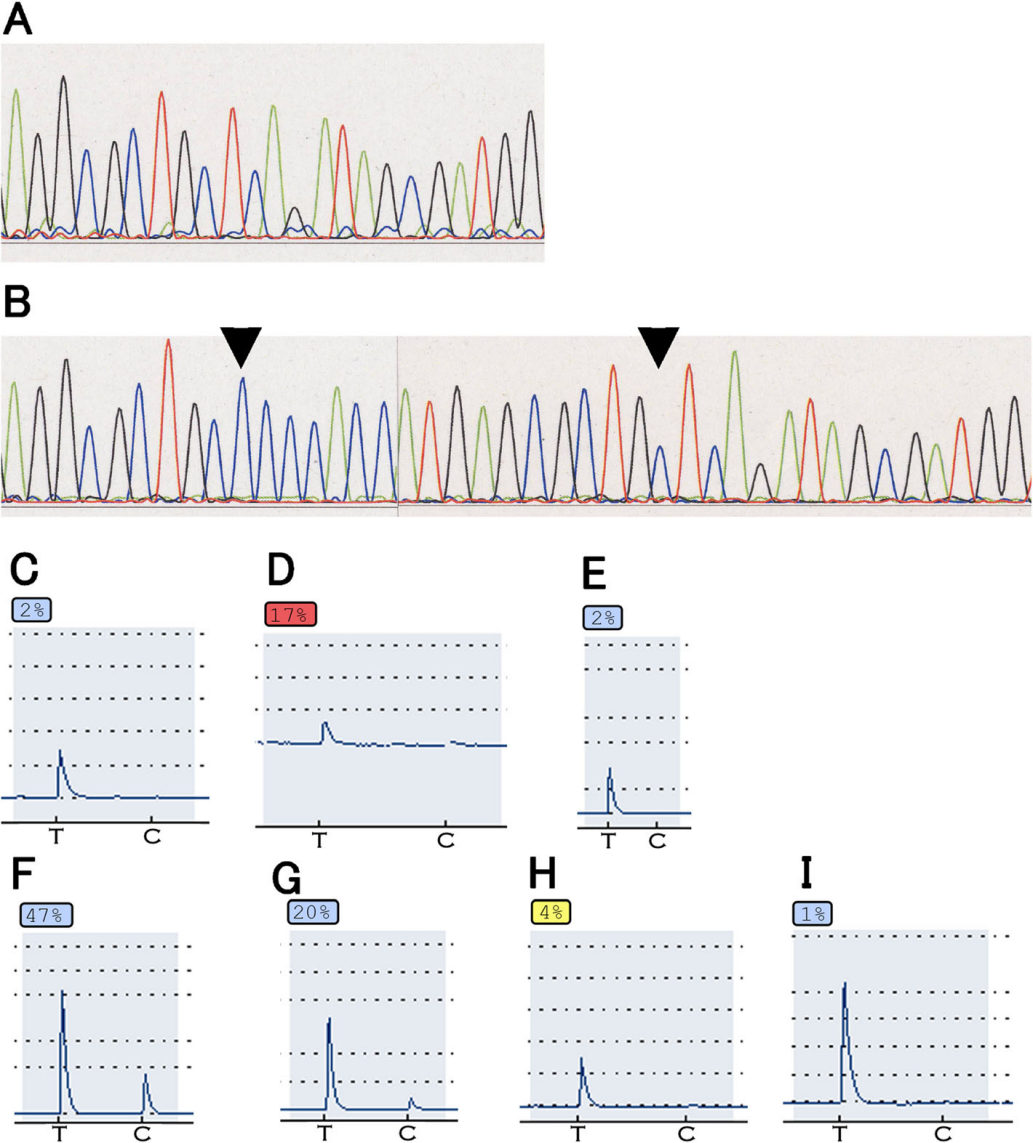
Molecular analysis of the representative case shown in Fig. 2. **a** In the CRA sample, a *TP53* deletion [c.529_546del18: deletion of codon 177_182 in exon 5, which is indicated between two arrowheads in the (**b**) normal sample] was seen; **c-h** DNA methylation analysis. **c** *LOX*, **d** *MINT31* and **e** *RUNX3* were not hypermethylated. In the second panel, hypermethylation was seen only for (**f**) *ELMO1* and not (**g**) *THBD* (**h**) *NEUROG1* or the *MLH-1* promoter (not shown), indicating that CRA can be classified as a low-methylated epigenotype

The results for molecular analyses are summarized in Tables 3 and 4, and results of *TP53* mutation and DNA methylation analysis of representative cases are shown in Fig. 3a-i. In addition, AI analysis of representative CRA mixed with poorly differentiated adenocarcinoma is shown in Supplementary Figure 1D-I. More AIs were detected in the poorly differentiated adenocarcinoma component than in CRA.

## Discussion

Several studies demonstrated that CRA was associated with specific histological patterns and had characteristic clinicopathological findings [3–7]. Despite its characteristic histological features, CRA is not included in the WHO classification as a distinct histological subtype. In histological analysis, this type of GC often displays characteristic tubules showing irregular (“crawling”) fusions, but pathologists nonetheless often incorrectly diagnose CRA as a benign non-neoplastic lesion, such as intestinal metaplasia. CRA may occur with low frequency in Western countries that have a low prevalence of *H. pylori* infection, which can produce intestinal metaplasia. Some recent studies indeed suggested that CRA could be considered as a specific histological entity of GC [3, 5], but to date CRA has not yet been histologically established as a recognizable subtype. CRA having low-grade cellular atypia may in fact closely mimic intestinal metaplasia. Based on promising data demonstrating peculiar clinicopathologic and molecular features of CRA, we suggest that identification of a CRA showing a “crawling” pattern be defined as a specific histological type in histological classification of GC.

The lace-like pattern might be a precursor type that can progress to diffuse type GC. We did not include CRAs having a lace-like pattern because these tumors are typically classified as diffuse type in histological diagnosis. Moreover, the lace-like pattern does not have the tubular structure that is recognized by pathologists as a basic histological feature of CRA [18]. Finally, the lace-like pattern is one of the representative histological findings of Epstein–Barr virus (EBV)-related GC, which is characterized by genome-wide enrichment in methylation and lack of MSI-high status at the molecular level [18]. We also excluded EBV-related cancer from the present study given that EBV-related cancer is an independent molecular phenotype in gastric molecular carcinogenesis.

Previous studies showed that GC has two histological types, intestinal and diffuse types [19]. CRA is classified as the intestinal type that has a characteristic tubular appearance in histological examinations [19]. Poorly cohesive (WHO classification)/signet ring cell carcinoma (SRCC) does not have a tubular structure. In addition, poorly cohesive/SRCC is commonly part of routine histological and pathological diagnoses in Japan. Therefore, poorly cohesive/SRCC was in a different histological group than CRA and was consequently excluded from the present study. In an earlier study, the frequency of CRA was reported to be 2.9% among primary early GCs [3] that were defined as intramucosal cancer or invasive gastric cancer that does not invade below the submucosa, irrespective of lymph node metastasis. We thus think that the frequency of CRA we found in the present study was not an underestimate of the actual frequency.

Mucin phenotype has been reported to be correlated with aggressiveness or genetic profiles of GCs. GCs with a gastric phenotype were characterized by allelic imbalance of 3p [20]. In addition, previous reports have shown that GCs with a gastric mucin phenotype were associated with poor survival [21]. Although some case series studies demonstrated that the mixed or intestinal mucin phenotype is a major type in CRAs [3. 7], there was no significant difference in the frequency of each mucin phenotype between the CRAs and CDAs in the present study. This finding suggests that the mucin phenotype in CRA and CDA shares a common mechanism in CRA pathogenesis.

Nuclear accumulation of β-catenin is frequently observed in human GCs [22, 23]. However, an overwhelming majority (98%) of CRA cases we examined were negative for nuclear β-catenin, whereas nearly one-third of CDA cases were β-catenin positive. This finding suggested that Wnt signal activation that results in nuclear accumulation of β-catenin plays only a minor role in CRA carcinogenesis. In addition, the present result might suggest that CRA and CDA activate Wnt signaling via different mechanisms.

Mutation of the *TP53* gene is an important genomic event in gastric carcinogenesis. A previous study showed that the frequency of *TP53* mutations varies according to histological type and tumor grade [24]. For example, *TP53* mutation frequently occurs in intestinal type adenocarcinoma, but is rarely seen for diffuse type adenocarcinoma [24], and the *TP53* mutation status can depend on tumor grade. *TP53* mutations are less frequent (10 ~ 20%) in intramucosal cancer relative to advanced cancers that have invaded beyond the submucosa [14, 24, 25]. In the present study, we found that *TP53* mutations are frequent in CRA having low grade atypia, compared with that for CDA. This finding suggests that *TP53* mutation plays a major role in carcinogenesis of CRA. In addition, the c.529_546 deletion in the *TP53* gene was not detected in CDA cases but was closely associated with carcinogenesis of CRA in this study. Although *TP53* mutation usually manifests as a missense mutation in human cancers, findings from this study suggested that *TP53* deletion mutants which result in negative expression of p53 characterize CRA tumorigenesis. and is, to our knowledge, the first study to characterize CRA in terms of a specific *TP53* gene mutation. Mutation of *TP53* was not correlated with p53 overexpression due to a deletion type mutation that resulted in negative p53 expression in the present study. According to this finding, we suggest that immunohistochemical tests for p53 cannot be substituted for sequencing of *TP53* mutations in CRA.

Allelic imbalance (AI) is an important genomic change that is an indicator of genomic instability. Previous studies showed that the presence of multiple AIs can be predictive of tumor aggressiveness in GCs [25, 26]. CRA can transform into a poorly differentiated component within the same tumor and invade into the submucosa without an accompanying increase in tumor grade [3, 4]. Multiple AIs identified in a CRA might explain these clinicopathologic findings for CRA. In the present study, CRAs often contained a poorly differentiated adenocarcinoma component. In addition, more AIs were detected in the poorly differentiated adenocarcinoma component than in the CRA component, indicating that CRA can progress to poorly differentiated adenocarcinoma. CRA is also characterized by low-grade nuclear atypia in which AIs are less frequent. In the present study, despite the low-grade atypia, we frequently observed multiple AIs in the CRA cases we examined. This finding of multiple AIs in CRAs is notable as it differs from the expectation that multiple AIs correlate with nuclear grade. Thus, these results could form an important basis for evaluation of gastric pathogenesis.

MSI is considered to be an important molecular event in gastric carcinogenesis [25] and is known to result from deficiencies in the activity of mismatch repair genes (MMR genes) [27]. In sporadic GCs, MSI is caused by DNA methylation of the *MLH-1* gene [27]. Inactivation of MMR genes alone is not a transforming event and additional genomic changes are needed to determine tumor progression [27]. MSI cancers are associated with 100- to 1000-fold increased mutation rates compared to microsatellite stable (MSS) tumors. Mutations in genes that regulate the cell cycle and apoptosis (e.g., *TGFβ RII*, *IGFIIR*, *TCF4*, *RIZ*, *BAX*, *CASPASE5*, *FAS*, *BCL10*, and *APAF1*) or maintain genomic integrity (e.g., *hMSH6*, *hMSH3*, *MED1*, *RAD50*, *BLM*, *ATR*, and *MRE11*) have also been associated with MSI-H GC [27]. The prevalence of GC with an MSI-high phenotype was previously shown to occur in 5–10% of sporadic GC cases, suggesting a relatively high frequency of a genomic phenotype in these types of GCs, which, in routine practice, is not a rare phenotype [25, 27]. In the present study, there were significant differences in the frequencies of MSI and *MLH-1* methylation between CRA and CDA, suggesting that MSI plays no significant role in CRA tumorigenesis. Moreover, CRA is not a candidate histological type of sporadic GC that has an MSI-high phenotype.

A recent study showed that CRA is characterized by *RHOA* mutation [27], which also frequently occurs in diffuse type GC [28]. The function of RhoA has been elucidated in previous studies [28–30]. First, RhoA is a critical regulator of actin–myosin-dependent cell contractility and cellular motility [28, 29]. As a result, *RHOA* signaling drives amoeboid motility that may be caused by protease-independent cellular movement [29–31]. This finding suggests that *RHOA* mutation plays a potential role in the diffuse invasive pattern of GC. Second, *RHOA* mutation has been reported to be harbored in intramucosal CRA having a poorly differentiated component, suggesting that *RHOA* mutation is an early event in gastric carcinogenesis [29]. According to this finding, *RHOA* mutation might enhance de-differentiation of CRA into a poorly differentiated component. Further functional study will be needed to identify the role of *RHOA* mutation in CRA.

In routine practice, we have encountered diffuse type cancer with a CRA phenotype at the mucosa. This finding suggests that CRA may be one type of precursor lesion that progresses to diffuse type GC. This finding might point to a link between CRA and SRCC/Por 2 (Japanese classification) that corresponds to poorly cohesive carcinoma/SRCC and diffuse type GC in WHO and Lauren’s classification, respectively.

DNA methylation plays a significant role in gastric carcinogenesis. The CpG island methylator phenotype (CIMP) characterizes distinct GC subtypes and the relationship between specific methylation patterns and molecular features has been evaluated [25, 32]. Here, LME was a distinct epigenetic pattern for CRA, compared with CDA that is associated with IME. This finding suggests that DNA methylation plays only a minor role in early CRA carcinogenesis. Finally, our results suggest that epigenetic alterations might be associated with different histological subtypes.

GCs have been divided into four molecular subtypes including MSI, EBV-related cancer, chromosomal instability (CIN) and genomically stable (GS) subtypes in comprehensive genomic analyses conducted by The Cancer Genome Atlas Research Network (TCGA) [33]. Although GC with an MSI phenotype and EBV-related GC have distinct molecular phenotypes, CIN and GS types are heterogeneous entities and thus not specific molecular subtypes. Although the CIN type is characterized by intestinal type, the GS type is histologically relevant to the diffuse type. If CRA that is classified into an intestinal type transforms into a diffuse type, the genomic phenotype will be expected to change into a GS type. However, a diffuse type transformed from a CRA might be a chromosomal instability type as supported by the finding that a high frequency of AIs is found in CRA. These findings are of interest despite the finding that CRA is transformed into a diffuse type at an advanced disease stage, and that the molecular pattern is similar to that of the intestinal type that shows chromosomal instability.

This study does have some limitations. First, we used only a limited number of markers to examine molecular alterations in CRA. More comprehensive genome- and epigenome-wide analyses have been performed to evaluate gastric carcinogenesis in recent reports [32], although the paraffin-embedded tissues used here, which likely contain fragmented DNAs, would hinder a similar comprehensive analysis. However, AI analysis of paraffin-embedded tissue samples may be suitable for examining genomic alterations. Second, the current results were not validated in a second cohort. Given that CRA is a relatively uncommon histological subtype in GC classification, more cases are needed to evaluate molecular alterations in a second cohort, and cases are currently being compiled.

## Conclusions

CRA is a type of GC that is characterized by distinct histological and molecular features. In addition, CRA was more frequently a depressed type compared to CDA. Nuclear accumulation of β-catenin and loss of MLH-1 expression were less frequent in CRA than in CDA. At a molecular level, frequent *TP53* mutation was closely associated with CRA pathogenesis. In particular, CRA is characterized by a c.529_546 deletion mutation in the *TP53* gene that is rarely seen for CDA. Finally, the presence of multiple AIs plays a major role in early carcinogenesis of CRA. On the other hand, DNA methylation accompanied by decreased expression of cancer-related genes likely has a less prominent role in CRA pathogenesis. Based on our findings, we suggest that CRA is an independent histological subtype of GC in terms of clinicopathological and molecular findings.

## Supplementary information

**Additional file 1: Figure S1.** A representative case with CRA transforming into poorly differentiated adenocarcinoma. (A) Intramucosal crawling-type adenocarcinoma (CRA) (on the right side) and poorly differentiated adenocarcinoma component (on the left side) can be observed in the hematoxylin and eosin (H&E) section. Histology of CRA (B) and poorly differentiated adenocarcinoma (C) components micro-dissected for molecular analysis. (D-F) Allelic imbalance of D22S1168 compared with normal mucosa (D); both CRA (E) and poorly differentiated adenocarcinoma (F) components showed loss of heterozygosity (LOH) (black arrows indicate reduction in first peaks with allele peak ratios of 0.45 and 0.47, respectively) (G-I) Allelic imbalance of D8S513 compared with normal mucosa (G), although CRA (H) showed heterozygosity (allele peak ratio of 0.98), poorly differentiated adenocarcinoma (I) showed LOH (black arrow indicates a reduction in the first peak with an allele peak ratio of 0.56).

## Abbreviations

CRA: Crawling-type adenocarcinoma
GC: Gastric cancer
CDA: Conventional differentiated adenocarcinoma
AI: Allelic imbalance
SSCP: Single strand conformation polymorphism
MSI: Microsatellite instability
HME: High methylation epigenotype
IME: Intermediate methylation epigenotype
LME: Low methylation epigenotype
SRCC: Signet-ring cell carcinoma
EBV: Epstein–Barr virus
CIMP: CpG island methylator phenotype
CIN: Chromosomal instability
GS: Genomically stable
