# Supplementary figures and images for: Analysis of clinicopathological and molecular features of crawling-type gastric adenocarcinoma
Source: Diagn Pathol. 2020 Sep 17;15:111. doi: 10.1186/s13000-020-01026-7 (PMC7500034; doi:10.1186/s13000-020-01026-7)

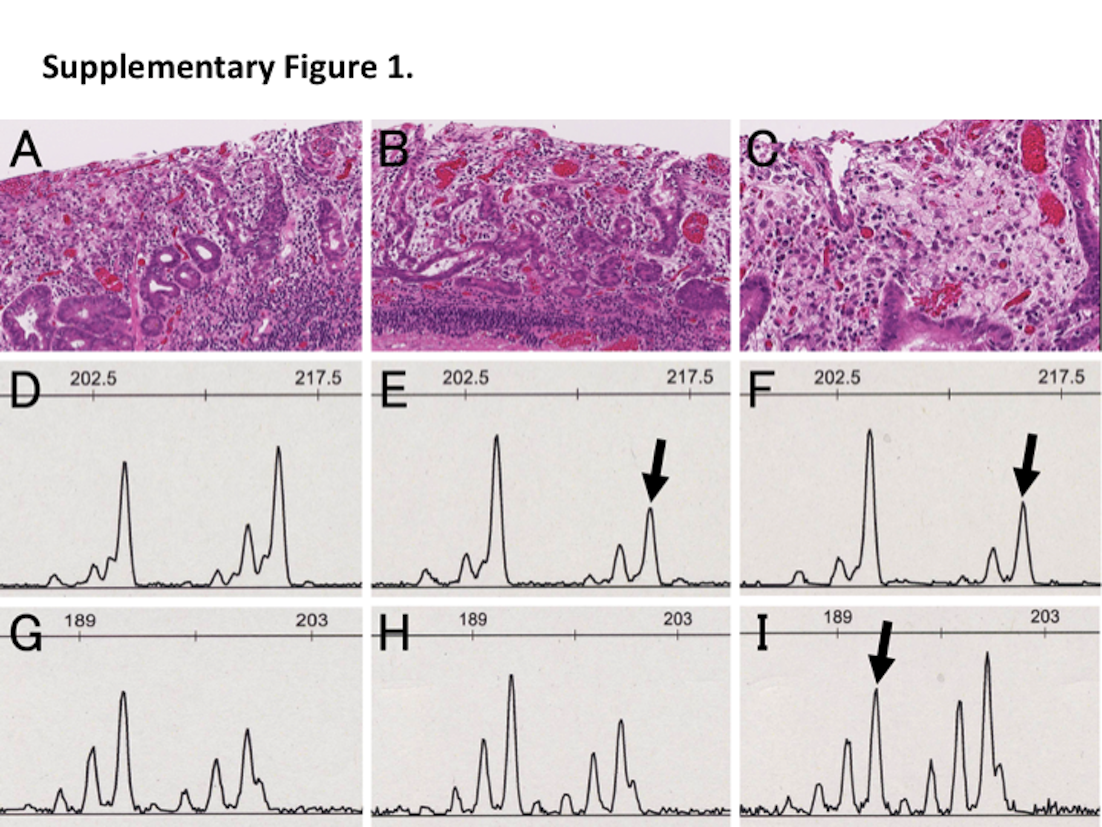

Supplement: Supplementary file 1 — Additional file 1: Figure S1. A representative case with CRA transforming into poorly differentiated adenocarcinoma. (A) Intramucosal crawling-type adenocarcinoma (CRA) (on the right side) and poorly differentiated adenocarcinoma component (on the left side) can be observed in the hematoxylin and eosin (H&E) section. Histology of CRA (B) and poorly differentiated adenocarcinoma (C) components micro-dissected for molecular analysis. (D-F) Allelic imbalance of D22S1168 compared with normal mucosa (D); both CRA (E) and poorly differentiated adenocarcinoma (F) components showed loss of heterozygosity (LOH) (black arrows indicate reduction in first peaks with allele peak ratios of 0.45 and 0.47, respectively) (G-I) Allelic imbalance of D8S513 compared with normal mucosa (G), although CRA (H) showed heterozygosity (allele peak ratio of 0.98), poorly differentiated adenocarcinoma (I) showed LOH (black arrow indicates a reduction in the first peak with an allele peak ratio of 0.56). [file 13000_2020_1026_MOESM1_ESM.tiff]
